# ﻿Caddisflies (Trichoptera) of Mongolia: an updated checklist with faunistic and biogeographical notes

**DOI:** 10.3897/zookeys.1111.76239

**Published:** 2022-07-11

**Authors:** Suvdtsetseg Chuluunbat, Bazartseren Boldgiv, John C. Morse

**Affiliations:** 1 Department of Biology, Mongolian National University of Education, Baga toiruu 14, Ulaanbaatar 14191, Mongolia Mongolian National University of Education Ulaanbaatar Mongolia; 2 Department of Biology, National University of Mongolia, Ikh surguuliin Gudamj 1, Ulaanbaatar 14201, Mongolia National University of Mongolia Ulaanbaatar Mongolia; 3 Department of Plant & Environmental Sciences, Clemson University, Clemson, SC 29634-0310, USA Clemson University Clemson United States of America

**Keywords:** East Palearctic, habitats, regional affinities, river sub-basin, species abundance, species diversity, species richness

## Abstract

To establish the biogeographic affinities of the caddisfly fauna of Mongolia, published records and results of our faunistic studies were analyzed. This study captured more than 47,000 adults collected from 386 locations beside lakes, ponds, streams/rivers, and springs in ten sub-basins of Mongolia using Malaise traps, aerial sweeping, and ultraviolet lights. In total, 201 species have been recorded, and approximately 269 species may occur in Mongolia according to our estimation. In a comparison of species richness for the family level, the Limnephilidae and Leptoceridae were the richest in species. The families Brachycentridae, Glossosomatidae, and Psychomyiidae had low species richness, but they included the most dominant species in terms of abundance and/or the percentage of occurrence in the samples from multiple sub-basins. Comparing the sub-basins, the Selenge had the highest Shannon diversity (H’ = 3.3) and the Gobi sub-basin had the lowest (H’ = 1.5). According to the Jaccard index of similarity, caddisfly species assemblages of Mongolia’s ten sub-basins were divided into two main groups: One group includes the Selenge, Shishkhed, Bulgan, Tes, and Depression of Great Lakes sub-basins; the other group includes the Kherlen, Onon, Khalkh Gol, Valley of Lakes, and Gobi sub-basins. The majority of Mongolian species were composed of East Palearctic taxa, with a small percentage of West Palearctic and Nearctic representatives and an even smaller percentage from the Oriental region, suggesting that the Mongolian Gobi Desert is, and has been, a significant barrier to the distribution of caddisfly species between China and Mongolia.

## ﻿Introduction

Mongolia is a large, land-locked country located in the southeastern East Palearctic Region (Morse 2021) for which knowledge of the freshwater fauna was poorly known. An understanding of a regional and local fauna is important for assessing ecosystem services and informing conservation management, especially for large areas with little faunistic research such as Mongolia. Survey efforts provide basic knowledge of faunal diversity within regional or local scales (Gelhaus et al. 2008; Heino 2009; Morse 2016) with cumulative diversity increasing as spatial and temporal scales of studies increase (Dodds 2002). Our long-term series of surveys for aquatic invertebrate diversity in Mongolia confirms these observations and expands the faunal and biogeographical knowledge of the country. Our four surveys occurred during 2002–2005 as the Hovsgol_GEF (Dynamics of biodiversity loss and permafrost melt in Lake Hovsgol, National Park, Mongolia), during 2003–2006 as the SRP (Selenge River Basin Project), during 2008–2011 as the MAIS (Mongolian Aquatic Insect Survey funded by US-NSF), and during 2016–2019 as the MACRO (Macroecological Riverine Synthesis funded by US-NSF) projects conducted as expeditions to study the aquatic insects in Mongolia.

Caddisflies (Trichoptera) constitute one of the major aquatic insect groups (Morse et al. 2019a). They are found in both lotic (streams and springs) and lentic (lakes, ponds, pools, and marshes) habitats (Wiggins 1996) and are great contributors to ecosystem functioning as shredding consumers of leaf litter (e.g., Limnephilidae), scrapers of periphyton (e.g., Apataniidae, Glossosomatidae, Psychomyiidae), filterers of suspended organic particles and tiny prey (e.g., Brachycentridae), and predators (e.g., Leptoceridae, Rhyacophilidae). In turn, they are an important component of the diet for fish and other invertebrates (Dodds 2002; Morse et al. 2019a, 2019b). Immature stages of caddisflies are well-studied and generally intolerant of environmental pollution, and thus, they are used as bioindicators in freshwater biomonitoring (Barbour et al. 1999; Dodds 2002).

The order Trichoptera includes more than 16,775 species belonging to 52 families in two monophyletic suborders, Integripalpia and Annulipalpia (Thomas et al. 2020; unpublished data). Trichoptera constitute the seventh most species-rich order of insects (Thomas et al. 2020). The fauna of the East Palearctic Biogeographic Region includes at least 1,244 species of caddisflies (Morse 2016; unpublished data).

The Trichoptera of Mongolia have been studied from the early 19^th^ century and were extensively investigated by foreign and Mongolian researchers through many expedition surveys, especially in the past 20 years (Chuluunbat et al. 2016). According to their investigations, 198 species have been recorded. For our checklist, we have reviewed 64 taxonomic publications which reported Mongolian caddisfly species and their distribution. We also include specimens collected and identified from our expeditions throughout the northern and western parts of the country from 2003 through 2011. The spatial distribution of species is reported and compared by provinces (or “aimags” in Mongolian), which is an administrative subdivision for the country and commonly reported and interpreted in previous publications (e.g., Chuluunbat et al. 2016).

In this study, we characterize caddisfly biogeographical distribution in ten major river basins and provide a revised and annotated checklist for the Trichoptera fauna in Mongolia. We assess the species richness and diversity of caddisflies in ten sub-basins (biogeographical regions), hypothesizing that they will be conspicuously different, and compare the similarities of species among the sub-basins and with the adjacent regions of neighboring countries.

## ﻿Materials and methods

### ﻿Study area

Mongolia is located in Central Asia, covering 1,564,118 km^2^. The area is characterized by an extreme continental climate with four distinct seasons including a long, cold, dry winter and short, hot summer; average annual precipitation is 220 mm (Natsagdorj 2014).

Mongolian surface water network is divided into three different major basins. The Mongolian northern Arctic Ocean Basin (**AOB**) contains the highest density or 52% of the country’s surface water network (Davaa 2015), including the following nine major rivers: the Orkhon, (the longest river in Mongolia), Ider, Tuul, Kharaa, Yoroo, Eg, Delgermurun, and Shishkhed Rivers, which are all tributaries of the Selenge River (Davaa and Oyunbaatar 2017); samples examined in this study were from all these rivers. The Yolt and two other streams are tributaries of the Hurimt River, which is a headwater of the Black Irtysh River (Shagdar 2006); no samples were taken from the Hurimt River itself. The AOB includes five major lakes: Hovsgol, Dood Tsagaan, Sangiin Dalai, Terkhiin Tsagaan, and Ugii.

The Central Asian Internal Drainage Basin (**CAIB**) covers a vast area from the western Altai Mountains to the eastern Dornod Steppe and 32% of the surface water network. It includes the following five major lakes: Uvs, Khyargas, Khar Us, Khar, and Airag. It also includes the following 11 rivers: the Khovd, Zavkhan, Baidrag, Buyant, Bulgan, Uyench, Bodonch, Sagsai, Ongi, Tes, and Tuin Rivers (Davaa 2015; Davaa and Oyunbaatar 2017); samples were collected from all these lakes and rivers.

The Pacific Ocean Basin (**POB**) contains 16% of Mongolia’s surface water network and includes the Kherlen, Onon, Ulz, Khalkh Gol, Numrug, and Degee Rivers; samples were from all six of these rivers. Kherlen River is the longest river in the basin and provides an inflow for Dalai Lake in China. The three major lakes are the Buir, Yakhi, and Khukh (Davaa and Oyunbaatar 2017).

According to Dulma (1979), Sokolov (1983), and Mendsaikhan et al. (2017), those three basins are further divided into nine sub-basins. However, their subdivisions were based entirely on the biogeography of fish distributions and does not include scattered water bodies in the Gobi that are without a fish fauna. Therefore, based on the distribution of aquatic beetles throughout Mongolia, ten regional sub-basins were proposed and published by Enkhnasan and Boldgiv (2019) by adding the Gobi sub-basin. These ten regional sub-basins include the Tes, Valley of Lakes, Depression of Great Lakes, and Gobi sub-basins in the CAIB; the Selenge, Shishkhed, and Bulgan in the AOB; and the Kherlen, Onon and Khalkh Gol sub-basins in the POB. According to Dulma (1979), Sokolov (1983), Mendsaikhan et al. (2017), and Enkhnasan and Boldgiv (2019), the divisions for hydrobiological studies suggest that the Bulgan River is in the AOB; in contrast, the hydrological classification by Davaa (2015) and others (Davaa and Oyunbaatar 2017) places the Bulgan River in the CAIB; we include the Bulgan River in the AOB.

### ﻿Database

The database was compiled from two main sources: caddisfly records published in papers cited by Chuluunbat et al. (2016) and caddisfly specimens collected by our own surveys and those kept in private collections (Prof. Bayartogtokh and Dr Puntsagdulam). In our previous publication (Chuluunbat et al. 2016), we used specimens collected during our own expeditions from 2003 through 2011. In this paper, additional specimens collected through 2020 and other personal collections were considered. That is to say, an enormous amount of species-level data collected by our long-term series of surveys (Hovsgol_GEF 2002–2005, SRP 2003–2006, MAIS 2008–2011, MACRO 2016–2019), preserved in private collections, and reported in previous publications since the early 20^th^ century were compiled in the current paper. One of our goals was to determine the estimated species richness; thus we needed species abundance data. Most of the early publications simply listed species without any individual numbers and without precise collection data due to lack of both precise positioning tools and standard transliteration of geographical names. We databased any species abundances reported in publications; however, if species were only listed without number of specimens, we counted the number of individuals as “one.” We realize that this procedure may have underestimated the abundance of these species, which in turn might overestimate species richness. In the literature sources, if species distribution or location information was provided without any details for an exact location, then we added the species records to our nearest collection sites that have geographical details. Non-verifiable records, not supported with voucher specimens, were omitted from the database.

**Figure 1.**
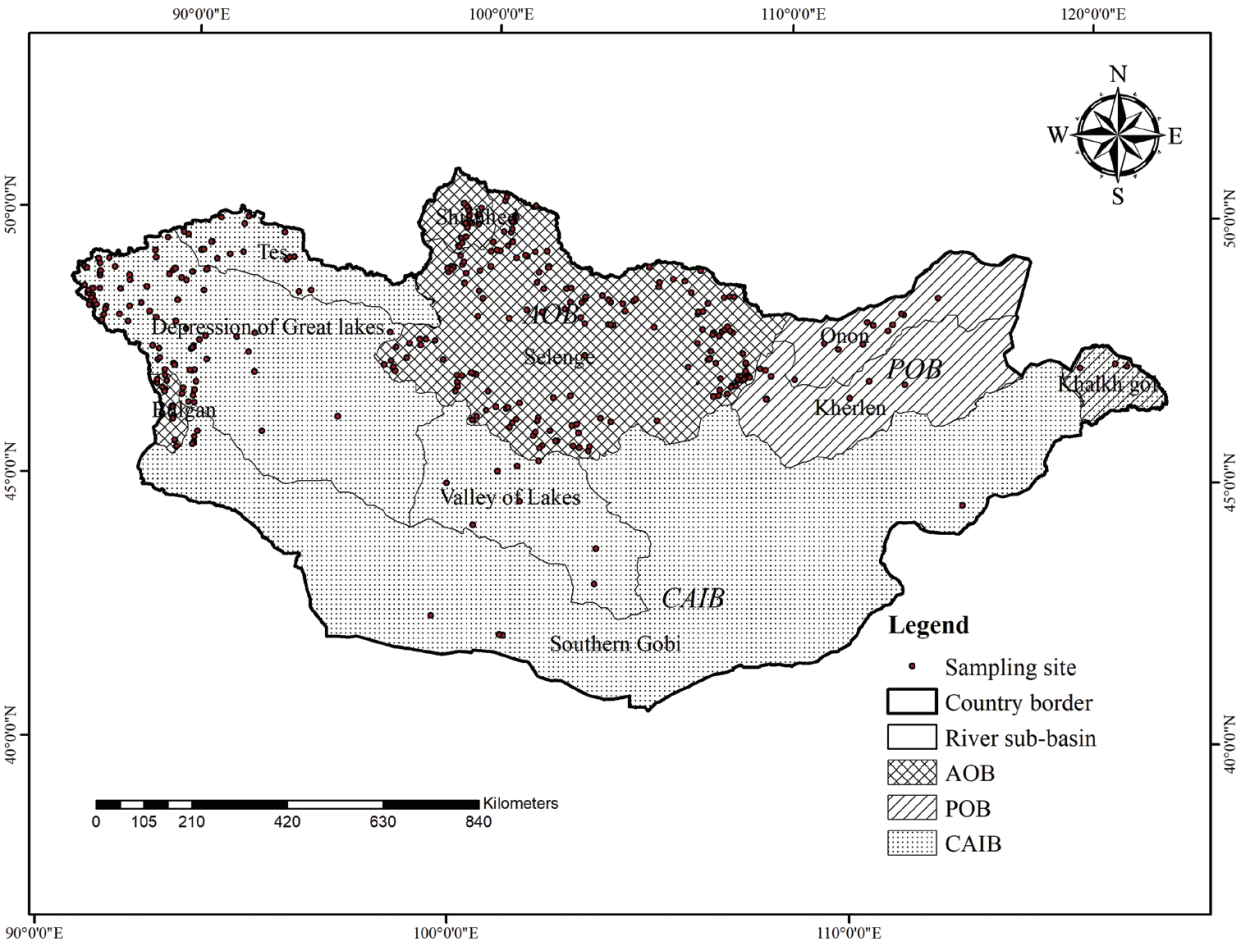
Mongolian watershed basins and ten sub-basins with 386 sampling sites. Abbreviations: AOB = Arctic Ocean Basin, CAIB = Central Asian Internal Basin, POB = Pacific Ocean Basin.

A total of 47,931 individuals from 386 sampling sites were databased as distributed in ten regional sub-basins (Fig. 1) and four different types of water bodies or habitats (lakes, ponds/pools, rivers/streams, and springs). The river/stream type represents 1^st^ to 7^th^ orders of streams and rivers (Table 1).

**Table 1.** Numbers of habitat types of water bodies sampled in ten sub-basins of Mongolia. Key: AOB = Arctic Ocean Basin, CAIB = Central Asian Internal Basin, POB = Pacific Ocean Basin.

No.	Sub-basins	Lake	Pond/Pool	River/stream	Spring	Total
1	Selenge (AOB)	18	7	152	14	191
2	Shishkhed (AOB)	4	3	9	2	18
3	Bulgan (AOB)	2	0	22	2	26
4	Tes (CAIB)	5	0	16	1	22
5	Depression of Great Lakes (CAIB)	16	2	65	7	90
6	Valley of Lakes (CAIB)	0	0	6	1	7
7	Kherlen (POB)	2	0	8	2	12
8	Onon (POB)	0	0	12	0	12
9	Khalkh Gol (POB)	1	0	2	0	3
10	Gobi (CAIB)	1	0	1	3	5
11	Total	49	12	293	32	386

We have followed the regional sub-basin classification of Enkhnasan and Boldgiv (2019), defining ten sub-basins in Mongolia. For the world geographical divisions we adopted the seven biogeographic regions of the Trichoptera World Checklist (Morse 2021).

To document similarities of species assemblages for the adjacent neighboring countries, we compared faunistic data for Russia by Ivanov (2011), for China by Yang et al. (2016), and for Kazakhstan by Smirnova et al. (2016). The Mongolian caddisfly fauna was also compared to adjacent regions including the Altay Mountains, Sayan Mountains, Pribaikalie Region, and Chita Region of Russia; the Xinjiang, Gansu, and Inner Mongolian regions of China; and the Irtysh and Balkhash-Alakol regions of Kazakhstan. Lake Baikal species in Russia are excluded from comparison analyses because of the high level of endemism in the lake. Thus, we have compared 754 species of caddisflies from the above nine regions of their respective three countries for similarity analyses.

### ﻿Sampling and identifications

In our surveys, we used various collecting techniques such as aerial nets, light traps when air temperature was above 10 °C with no wind (McCafferty 1981), and two Townes-style Malaise traps (Townes 1972) placed at the edge of the water, one on bare ground and the other in tall grass or bushes. Malaise traps were placed for the duration of a week for Hovsgol_GEF samples in the rivers of the eastern shore of Lake Hovsgol, 12 hours for SRP and MAIS samples, and two hours for MACRO samples. Adult caddisfly identifications were accomplished under dissecting microscopes, using identification keys by Lehr (1997), Malicky (2004), and other authors. Verification of determinations for the most common 95 caddisfly species belonging to 41 genera and 13 families was accomplished through comparisons of their mtCOI barcodes with those of sequenced species from other countries maintained at the Canadian Centre for DNA Barcoding, Biodiversity Institute of Ontario, University of Guelph, under the Trichoptera Project of the Barcode of Life Database (BOLD Systems 2013). Sequences of the mtCOI gene for the 95 sequenced species in our studies are recorded in GenBank (Zhou et al. 2016).

### ﻿Statistical analysis

An abundance-based species accumulation curve was used to predict rarified species richness. Chao 1 was used as an estimator to show the relationship of sample sizes and numbers of species. EstimateS 9.1.0 software was used to calculate the Chao1, and 100 runs were performed to see the singletons (**S1**, one specimen of a species), doubletons (**S2**, two specimens of a species), and unique species (**SU**, species occurring at only one site) (Colwell 2013) at each collection location. Shannon’s index of diversity (***H***’) (Shannon and Weaver 1949), evenness (***J***’) (Pielou 1966), and Berger-Parker dominance index (***Dd***) (Berger and Parker 1970) were calculated for the ten sub-basins and for the country. Similarity of assemblages among the sub-basins was determined based on presence-absence data quantified by the Jaccard index method using Ward distance with the vegan package of R3.6.1 software (R Development Core team 2010).

## ﻿Results

**Figure 2.**
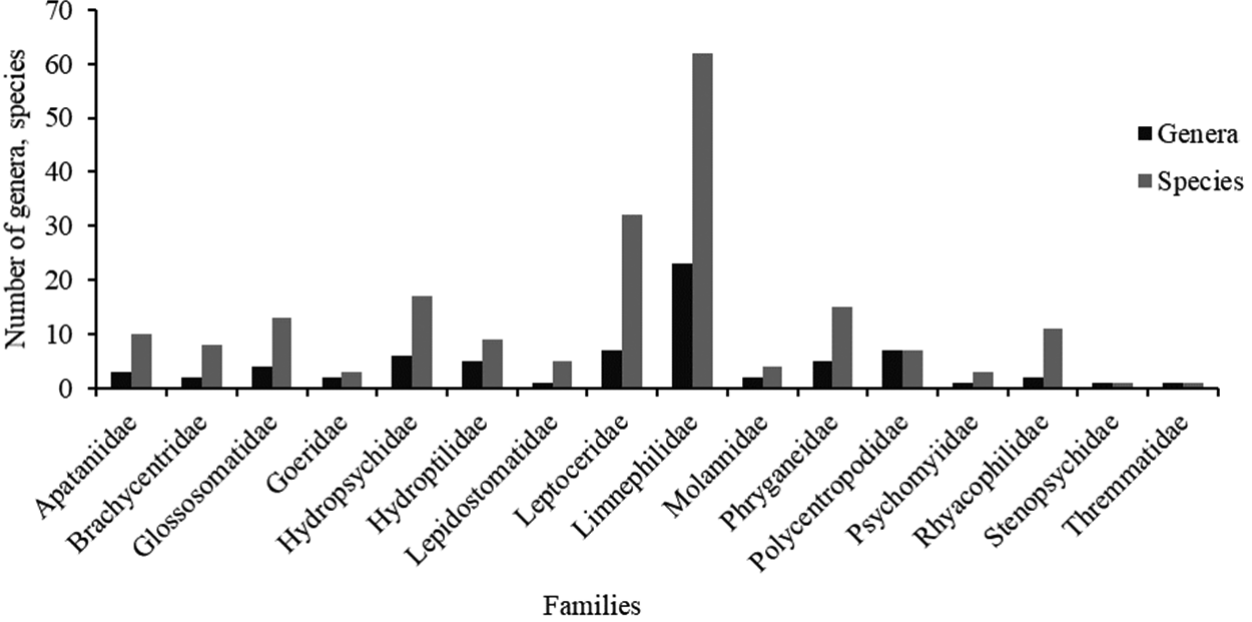
Number of genera and species of caddisfly families in Mongolia.

Based on the results of our data mining (species data from previously published literature) and our survey investigations, we found 201 caddisfly species representing 72 genera and 16 families in Mongolia (Appendix). Families with the most diverse genera and species were Limnephilidae (23 genera, 62 species) and Leptoceridae (7, 32); families with the least diverse genera and species were Psychomyiidae (1, 3), Goeridae (2, 3), Thremmatidae (1, 1), and Stenopsychidae (1, 1) (Fig. 2). The genera with the highest number of species were *Limnephilus* (25 species), *Ceraclea* (11), *Rhyacophila* (10), *Hydropsyche* (9), *Apatania* (8), *Agrypnia* (7), and *Glossosoma* (6) (Fig. 2, Appendix). In terms of abundance, families Brachycentridae and Psychomyiidae were most abundant (Table 2).

**Table 2.** Richness and diversity measurements of caddisflies for Mongolia and its ten sub-basins. Key: Sub-basins = sub-basin names, Sites = collection sites, N = number of individuals, Sobs = observed number of species, Chao1 = estimated number of species, S1 = singleton species, S2 = doubleton species, SU = unique species, *H*’ = Shannon-Weaver diversity index, *J*’ = Pielou’s evenness, *Dd* = Berger-Parker dominance index (by percentage of dominant species), dominant species for the sub-basin and Mongolia. AOB = Arctic Ocean Basin, CAIB = Central Asian Internal Basin, POB = Pacific Ocean Basin.

No.	Sub-basins	Sites	N	Sobs	Chao1	S1	S2	SU	*H*’	*J*’	*Dd*	Dominant species
1	Selenge (AOB)	191	19287	157	211	46	10	61	3,33	0,65	16%	*Rhaycophilaegijnica*
2	Shishkhed (AOB)	18	1635	51	63	13	5	25	2,48	0,63	30%	*Apataniamajuscula*
3	Bulgan (AOB)	26	4999	39	54	13	1	16	2,36	0,64	21%	*Psychomyiaminima*
4	Tes (CAIB)	22	2243	52	75	20	7	29	2,29	0,57	31%	*Brachycentrusamericanus*
5	Depression of Great Lakes (CAIB)	90	17306	88	110	16	4	25	2,57	0,57	28%	*Psychomyiaflavida*
6	Valley of Lakes (CAIB)	7	215	15	22	2	3	9	1,79	0,66	48%	*Brachycentrusamericanus*
7	Kherlen (POB)	12	1357	38	45	12	8	22	1,81	0,49	38%	*Paduniabikinensis*
8	Onon (POB)	12	719	34	43	12	6	18	1,77	0,5	54%	*Paduniabikinensis*
9	Khalkh Gol (POB)	3	64	17	21	8	5	11	2,26	0,79	22%	*Oecetisochracea*
10	Gobi (CAIB)	5	106	7	11	1	0	7	1,52	0,78	34%	*Colpotauliusincisus*
11	Mongolia	386	47931	201	269	53	16	69	3,38	0,63	16%	*Psychomyiaflavida*

Species diversity (*H*’) was highest in the Selenge River sub-basin (3.33) and the Depression of Great Lakes sub-basin (2.57), while evenness (*J*’) was highest in the Khalkh Gol sub-basin (0.79) and the Gobi sub-basin (0.78) (Table 2).

Species in families Brachycentridae, Glossosomatidae, and Psychomyiidae were most abundant (Table 2, Appendix). *Brachycentrusamericanus* (Banks, 1899) was the most dominant species in the Tes (31%) and Valley of Lakes (48%) sub-basins, with both of these sub-basins belonging to the CAIB. *Paduniabikinensis* Martynov, 1934, was the dominant species in the Kherlen and Onon sub-basins (38% and 54%, respectively), both belonging to the POB; and *Psychomyiaflavida* Hagen 1861 was the dominant species in the Depression of Great Lakes sub-basin (28%) and all of Mongolia (16%). Other species were dominant in the other five sub-basins (Table 2).

Abundance-based species accumulation analysis estimated that species of caddisflies occurring in Mongolia is 269 (Table 2). Among the 201 currently recorded species, 53 were represented by a single specimen at some sites (S1) and 16 were represented by two specimens at some sites (S2). In our study, 69 species occurred uniquely at a single site in Mongolia (SU) (Table 2).

**Figure 3.**
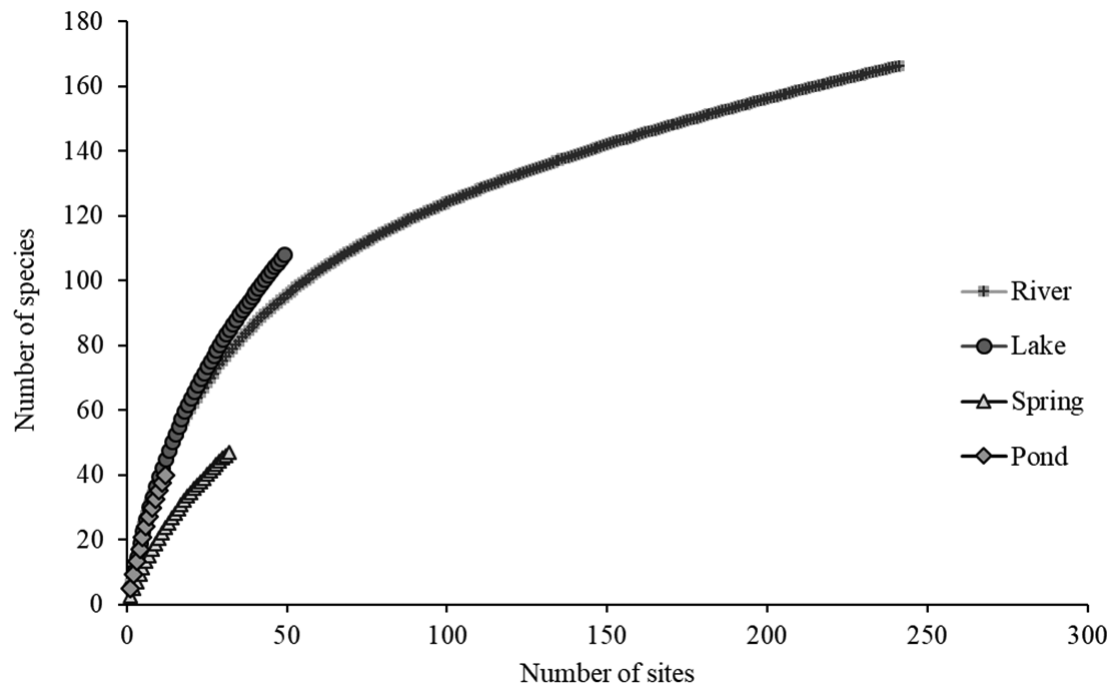
Caddisfly species richness collected from different numbers of sites from four different types of water bodies.

Caddisfly species richness varied greatly among the four different habitat types of water bodies. From 386 sampling sites, the highest species numbers (178 species) were from the various types of rivers. The next most-diverse habitat was lakes with 106 species. Springs and ponds were inhabited by 47 and 40 species, respectively (Fig. 3).

**Figure 4.**
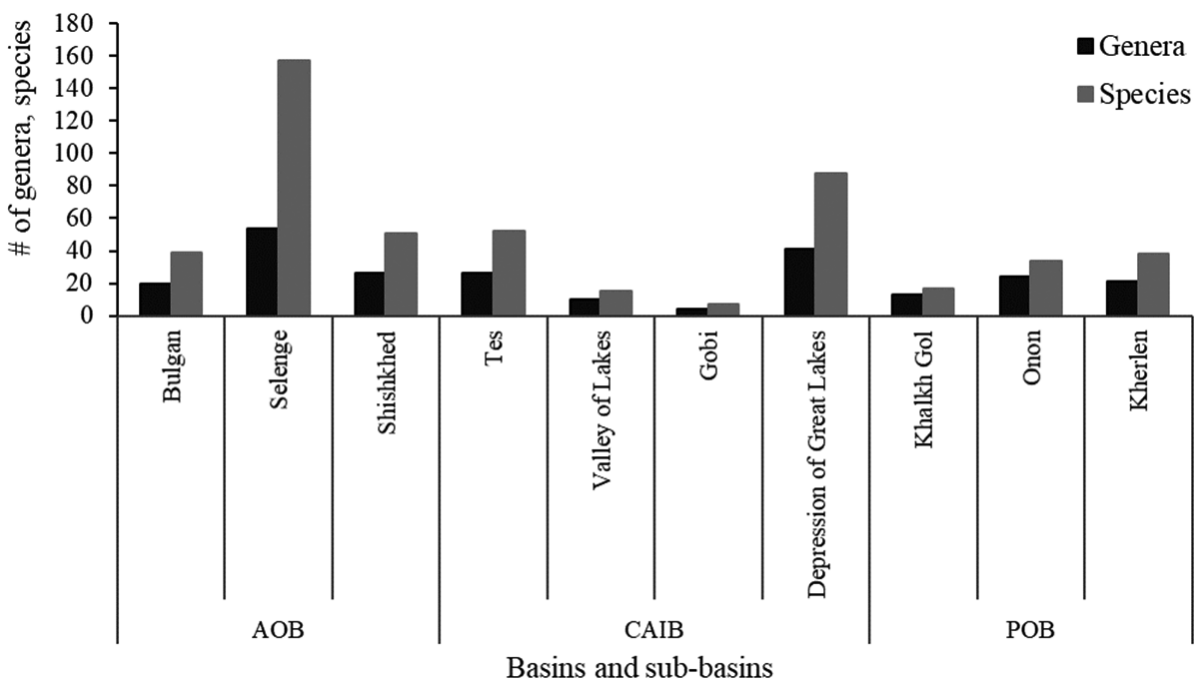
Richness of genera and species of caddisflies distributed in ten sub-basins of Mongolia. Abbreviations: AOB = Arctic Ocean Basin, CAIB = Central Asian Internal Basin, POB = Pacific Ocean Basin.

The regional distribution and richness of caddisflies in Mongolia varied in the ten sub-basins, ranging from 7 to 157 species. The highest numbers of species and genera of caddisflies occur in the Selenge River sub-basin (157 species, 54 genera), followed by the Depression of Great Lakes (88 species, 41 genera), the Tes and Shishkhed River sub-basins (50 and 49 species, respectively, in 26 genera), the Bulgan River sub-basin (39 species in 20 genera), Kherlen River sub-basin (38 species in 21 genera), the Onon River sub-basin (34 species in 24 genera), Khalkh Gol (17 species in 13 genera), the Valley of Lakes sub-basin (15 species in 10 genera), and the sub-basin with the lowest species richness was the Gobi sub-basin (7 species in 4 genera) (Table 2, Fig. 4).

**Figure 5.**
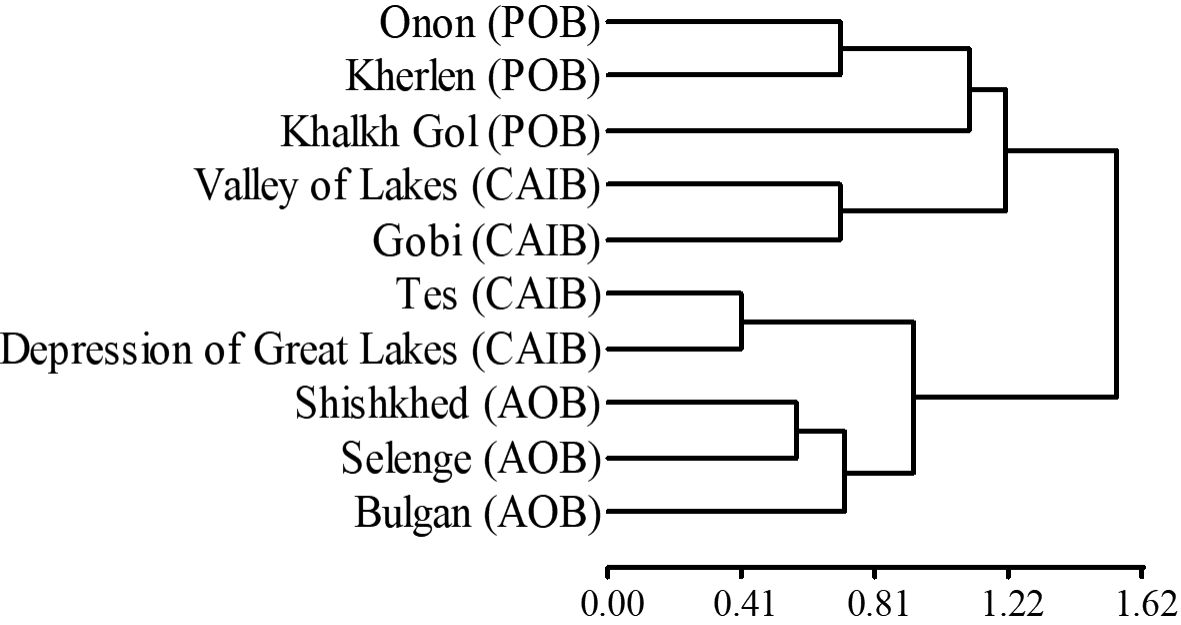
Similarities of caddisfly assemblages among ten sub-basins. Abbreviations: AOB = Arctic Ocean Basin, CAIB = Central Asian Internal Basin, POB = Pacific Ocean Basin.

Based on the distribution of 201 species of caddisflies in the ten sub-basins of Mongolia, similarities of caddisfly assemblages among sub-basins are shown in Fig. 5. The Onon, Kherlen, and Khalkh Gol sub-basins (POB) were more similar to the CAIB’s Valley of Lakes and Gobi sub-basins as one cluster, whereas the Shishked, Selenge, and Bulgan river sub-basins (AOB) were similar to the CAIB’s Tes and Depression of Great Lakes sub-basins in another group. The interesting results of this clustering showed that the CAIB’s sub-basins were divided into two clusters. The Valley of Lakes and Gobi sub-basins were more similar to those of the POB, the Tes and Depression of Great Lakes sub-basins were more similar to those of the AOB. That is, the caddisfly assemblages in the AOB and the POB were most dissimilar geographically, with the CAIB partially similar to each of them (Fig. 5).

**Figure 6.**
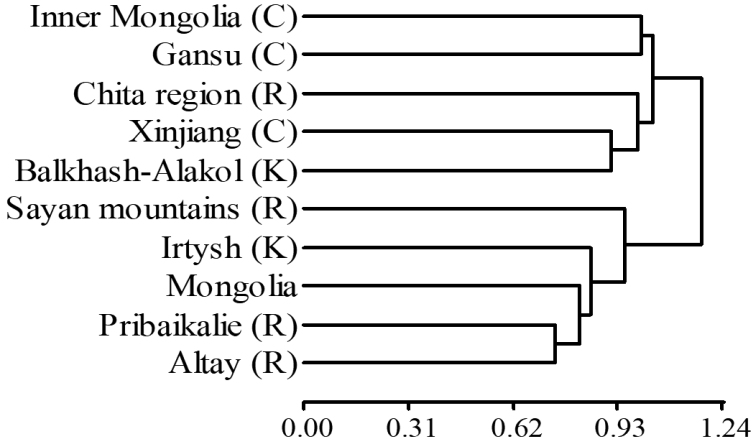
Caddisfly fauna similarities among Mongolia and nine adjacent regions of China (C), Kazakhstan (K), and Russia (R).

China, Kazakhstan, and Russia are large countries bordering Mongolia on the south, west, and north, respectively. To assess similarities with these surrounding countries, we selected their closest regions. Species assemblages for the neighboring regions were clustered into three groups. The first group was composed of Chinese Gansu and Inner Mongolia. The second group was composed of Russian Chita region and Kazakhstan’s Balkhash-Alakol and Chinese Xinjiang region. Finally, Russian Pribaikalie, Altay, and Sayan Mountains, Kazakhstan’s Irtysh basin, and Mongolia were clustered into one group (Fig. 6). Mongolian caddisfly species were most similar to those of the Russian and Kazakhstan faunas and least similar to the Chinese fauna (Fig. 6).

**Figure 7.**
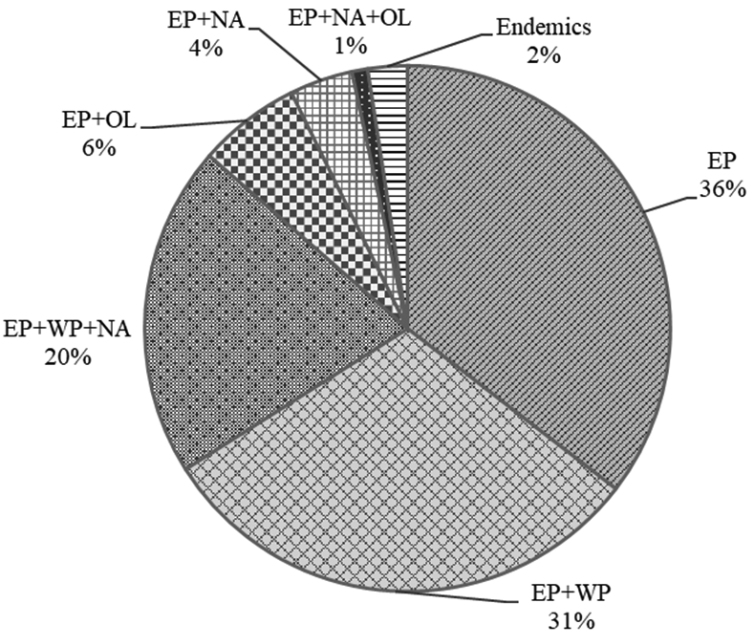
Mongolian caddisfly species composition according to world biogeographic regions (Morse 2021): East Palearctic (EP), Nearctic (NA), Oriental (OL), and West Palearctic (WP).

Most caddisfly species of Mongolia also inhabit other parts of the East Palearctic Biogeographic Region (98%). Among those, 31% occur also in Europe and northern Africa (WP). Another 20% of the Mongolian species are Holarctic, occurring also in the Nearctic and West Palearctic Regions. Six percent of the Mongolian-East Palearctic species occur also in the Oriental Region, 4% occur also in the Nearctic, and 1% occur in all three of these latter regions (Fig. 7). Caddisfly endemism is very rare for Mongolia (Fig. 7). Endemic species (**) and new country reports (*) are highlighted in the species list (Appendix). The list includes the following five new records for the country:

1. *Asynarchussachalinensis* Martynov, 1914: Tov Province, Erdene Soum, Upper Tuul River, 47.94981°N, 107.45511°E, elev. 1593 m, 2004.vi.29. coll. MAIS team, 1 male, det. S. Chuluunbat.
2. *Cheumatopsycheinfascia* Martynov, 1934: Dornod Province, Khalkh Gol, 31 km east of from Khalkh Gol Soum, 47.52556°N, 118.98538°E, elev. 736 m, 2006.vi.22, coll. J. Puntsagdulam, D. Altanchimeg, 2 males, 11 females, det. S. Chuluunbat.
3. *Oecetisnigropunctata* Ulmer, 1908: Khentii Province, Batnorov Soum, Bayanbulag, 47.91561°N, 111.50480°E, elev. 1328 m, 2020.vi.28, coll. B. Bayartogtokh, light trap, 1 male, 4 females, det. S. Chuluunbat.
4. *Drusus* sp.: Khovd Province, Duut Soum, Tsagaan Burgas Gol, 47.55936°N, 91.76095°E, elev. 1865 m, 2008.vii.15, coll. MAIS team, 7 males, 2 females, det. S. Chuluunbat.
5. *Nyctiophylax* sp.: Uvs Province, Zuungobi Soum, Nariin Gol, 50.05245°N, 94.15410°E, elev. 923m, 2009.vii.22, coll. MAIS team, 1 male, det. S. Chuluunbat.

## ﻿Discussion

Survey investigations contribute to knowledge of a regional fauna. Up to the late 1900s and early 2000s, Mongolian Trichoptera were investigated mostly by foreign scientists who recorded 129 species (Morse et al. 2006). Starting in early 2000, regional scientific expeditions surveyed various parts of Mongolia intensively and expanded the caddisfly species list up to 198 (Chuluunbat et al. 2016). We are currently adding five new country records to this list. Two subspecies (*Limnephilusextricatussibiricus* Mey, 1991, is a subspecies of *Limnephilusextricatus* McLachlan, 1865; *Phryganeagrandisrotundata* Ulmer, 1905, is a subspecies of *Phryganeagrandis* Linnaeus, 1758) are not shown as distinct taxa in the Mongolian species list (Appendix) and remain uncounted as species by Morse (2021). These findings result in a list of 201 species in Mongolia. This total number of documented species relative to the estimated 269 species indicates that (a) more survey work is needed in Mongolia to fully document the country’s caddisfly fauna and (b) the estimated number of species may have been overestimated due to lack of precise abundance data for some species as reported in previous literature.

A new synonym and changes in two species names are also reflected and updated from the list of Chuluunbat et al. (2016). *Stenopsychemarmorata* Navás, 1920, is a synonym of *Stenopsychegriseipennis* McLachlan, 1866, according to Kuranishi & Tanida (2016). *Micropternasequax* McLachlan, 1875, was reported as *Stenophylaxsequax* (McLachlan, 1875); *Synagapetusinaequispinosus* (Schmid, 1970) was reported as *Agapetusinaequispinosus* (Schmid, 1970) by Chuluunbat et al. (2016).

Different numbers of endemic species from Mongolia have been reported. By 2006, a single endemic species was reported in Lake Hovsgol, *Limnephilushovsgolicus* Morse, 1999 (Morse et al. 2006; Chuluunbat et al. 2016). However, Puntsagdulam et al. (2017) reported 7 endemic species of caddisflies for Mongolia: *Agapetusinaequispinosus* (Schmid, 1970); *Neureclipsismongolica* Schmid, 1968; *Rhyacophilaegijnica* Schmid, 1968; *Triaenodeskaszabi* Schmid, 1968; *Hydroptilapectinifera* Schmid, 1970; *Apatanianaimpexa* Schmid, 1968; with *Limnephilushovsgolicus*. However, *Agapetusinaequispinosus* (Schmid, 1970) is now acknowledged as *Synagapetusinaequispinosus* (Schmid, 1970) and has been reported from Russia (Ivanov 2011) and Japan (Kuranishi & Tanida 2016). *Neureclipsismongolica* Schmid, 1968, is a synonym of *Neucentropusmandjuricus* (Martynov, 1907) and has been reported also from China (Yang et al. 2016). *Rhyacophilaegijnica* Schmid, 1968, has been reported from Russia (Ivanov 2011). *Triaenodeskaszabi* Schmid, 1968, is a synonym of *Triaenodesjakutanus* Martynov, 1910, and reported from Russia (Ivanov 2011) and North America (Manuel 2010). *Hydroptilapectinifera* Schmid, 1970, has not been reported from any other country yet and the type locality is the Delgermurun River, Burenkhaan Soum (current administrative name, Burentogtokh Soum), Hovsgol Province in Mongolia (Schmid 1970). *Apatanianaimpexa* Schmid, 1968, has been reported from Russia (Ivanov 2011) and China (Yang et al. 2016). *Limnephilushovsgolicus* Morse, 1999, is endemic to Lake Hovsgol (Morse 2021). Also, *Agrypniahayfordae* Morse & Chuluunbat, 2007, has not yet been reported from other countries; it inhabits lakes and the type locality is Nuuriin Khooloi Lake, Thenkher Soum, Arkhangai Province in Mongolia. This species was collected also from Lake Terkhiin Tsagaan, Tariat Soum, Arkhangai Province, by colleagues in 2018. In conclusion, only three of the above species, i.e., *Hydroptilapectinifera*; *Limnephilushovsgolicus*; and *Agrypniahayfordae*, are the known caddisfly endemics for Mongolia (Appendix).

Distribution and diversity of Mongolian caddisflies are usually reported in the literature for the three main basins and administrative provinces or rivers rather than the ten sub-basins discussed here. Higher richness of caddisfly species was observed in the Arctic Ocean Basin (AOB) than in the other two basins by Dulmaa and Nansalmaa (1970). Our results reflect the same observation due to the fact that the same literature sources were used and higher sampling efforts (sites) by our own surveys occurred in the AOB than in the CAIB and POB. Strangely, higher species richness was observed in areas with a high density of water networks of rivers and lakes. The AOB and CAIB have a greater density of surface water networks than the POB, and a higher percentage (over 40%) of Mongolia’s geological formations including mountainous areas (Yembuu 2020) and isolated drainages. The AOB has higher stream connectivity than the CAIB, and the connectivity allows it to share similar species in the connected waterways, which tends to make the AOB to have lower species richness than the CAIB (Maasri et al. 2018). Despite this trend, the greater sampling effort of our surveys in the AOB has resulted in a higher species richness in the AOB than in the CAIB.

The highest species number was found in the family Limnephilidae, especially the genus *Limnephilus*. The genus *Limnephilus* is one of the largest genera with at least 185 species (unpublished data), inhabiting primarily cold water in northern latitudes and often found at higher altitudes (Ruiter 1995). These case-making caddisflies are known to be highly diverse and occur throughout the Holarctic Region (Morse 2016). Indeed, most of the species observed in Mongolia are case-making caddisflies (Apataniidae, Brachycentridae, Glossosomatidae, Goeridae, Hydroptilidae, Limnephilidae, Lepidostomatidae, Leptoceridae, Molannidae, and Phryganeidae). The elevated landscape of central and western Mongolian is especially suitable habitat for case-making and cold-water-dwelling caddisflies.

Among all types of habitats that were sampled, most of the species were observed in streams/rivers. The immature stages of most caddisflies can inhabit many available substrates in running water and are generally most diverse in streams and rivers (Resh and Rosenberg 1984); however, they are rare in springs (Thorp and Rogers 2011). The number of species occurring in lakes is relatively higher than those in ponds and springs; the 108 species observed from 48 lake sites indicate that lake-inhabiting Trichoptera have been investigated well in Mongolia.

The Mongolian Great Gobi Desert appears to represent an enormous barrier to distribution of caddisflies to and from the south. This pattern suggests a reason for the higher species richness observed in northern sub-basins (Selenge, Shishkhed, Bulgan, Tes, Depression of Great Lakes, Valley of Lakes, Kherlen, Onon and Khalkh Gol) than the Gobi sub-basin. Also, this might be the reason that caddisfly assemblages of Mongolia are more dissimilar to those in Chinese regions than to those in the Russian and Kazakh regions selected for comparison in this study. These results corroborate research indicating that more Mongolian caddisfly species are shared with Russia (Ivanov 2011) than with China (Yang et al. 2016). The composition of Mongolian caddisfly species and the low level of endemism we report here appear to be explained by similar biogeographic and meteorologic conditions in these and neighboring eastern, northern and western regions and the relatively dry, mostly inhospitable Gobi in southern Mongolia and northern China, resulting in a formidable isolation for aquatic insects (and possibly all freshwater biota) from the south for over 70 million years (Dashzeveg et al. 2005).

The similarity in the caddisfly species composition among the three main basins was not as different as we expected. The caddisfly assemblages in sub-basins of AOB and POB were different, but the CAIB was divided into two sub-basins more similar to either AOB or POB. This is probably due to the fact that the CAIB covers a large area from west to east in Mongolia. The faunas of the Depression of Great Lakes and Tes sub-basins of the CAIB in the northwest are more similar to those of the AOB, while the faunas of the Valley of Lakes and Gobi sub-basins of the CAIB in the south are more similar to those of the POB in eastern Mongolia, suggesting that Mongolian caddisfly species might be distributed differently than the faunas that were the basis of the current basin classification. In conclusion, the caddisfly fauna of Mongolia was investigated thoroughly, from the view of the distribution of species in different spatial scales with documented and estimated richness. Most of the species distributed in Mongolia are characteristic of the Palearctic Region. The caddisfly fauna of Mongolia was similar to Russia’s closest bordering regions of Altay and Sayan Mountains, Pribaikalie, and Kazakhstan’s Irtysh Basin, but different from that of China’s bordering regions due to the lack of connections of the surface water network and the presence of the Mongolian Gobi Desert. Sampling effort results in higher richness; thus, further sampling in the sub-basins especially in the Gobi may yield more species. Knowing the species richness in the basins, and sub-basins allow us to manage and protect aquatic systems better and provide necessary knowledge for future freshwater biomonitoring.

## ﻿Appendix

**Table A1.** Compiled caddisfly species list from our own data and taxonomic literature, and their distribution in basins and sub-basins of Mongolia. Key: 0 = not reported, 1 = reported. Females of some species (sp.) could be identified only to genus. * = new records for the country, ** = endemic species. Biogeographic regions: EP = East Palearctic, HL = Holarctic (EP+NA+WP), NA = Nearctic, OL = Oriental, WP = West Palearctic.

#	**Species name**	**Biogeographic regions**	**Arctic Ocean Basin**			**Pacific Ocean Basin**			**Central Asian Internal Basin**				**Total occurrence in the basins**	**Total abundance of species**
			**Selenge**	**Shishkhed**	**Bulgan**	**Kherlen**	**Onon**	**Khalkh Gol**	**Tes**	**Depression of Great Lakes**	**Valley of Lakes**	**Gobi**		
Apataniidae														
1	*Allomyiasajanensis* Levanidova, 1967	EP	0	1	0	0	0	0	0	0	0	0	1	9
2	*Apataniacrymophila* McLachlan, 1880	HL	1	1	0	0	0	0	0	0	0	0	2	41
3	*Apataniadalecarlica* Forsslund, 1942	EP+WP	1	1	1	0	0	0	1	1	0	0	5	89
4	*Apataniadoehleri* Schmid, 1954	EP	1	1	0	0	0	0	1	1	1	0	5	87
5	*Apataniamajuscula* McLachlan, 1872	EP+WP	1	1	1	1	0	0	1	1	0	1	7	2618
6	*Apataniamongolica* Martynov, 1914	EP	0	0	0	0	0	0	0	0	0	1	1	3
7	*Apataniastigmatella* (Zetterstedt, 1840)	HL	1	1	1	0	0	0	0	1	1	0	5	630
8	*Apataniasubtilis* Martynov, 1909	EP+WP	0	0	0	0	0	0	1	0	0	0	1	1
9	*Apataniazonella* (Zetterstedt, 1840)	HL	1	1	1	1	0	0	1	1	0	0	6	57
10	*Apatanianaimpexa* Schmid, 1968	EP	0	0	1	0	0	0	1	1	0	0	3	662
Brachycentridae														
11	*Brachycentrusamericanus* (Banks, 1899)	HL	1	1	1	1	0	0	1	1	1	0	7	6751
12	*Brachycentrusjaponicus* (Iwata, 1927)	EP	1	0	0	0	0	0	0	1	0	0	2	2
13	*Brachycentruskozlovi* Martynov, 1909	EP+OL	1	0	0	0	1	0	0	0	0	0	2	2
14	*Brachycentrussubnubilus* Curtis, 1834	EP+WP	1	0	1	0	0	0	0	0	0	0	2	6
15	*Brachycentrustazingolensis* Mey, 1980	EP	0	0	0	0	0	0	0	0	1	0	1	1
16	*Brachycentrusugamicus* Grigorenko & Ivanov, 1990	EP	1	0	0	0	0	0	0	0	0	0	1	1
17	Micrasema (gelidum) gelidum McLachlan, 1876	HL	1	0	0	1	0	0	0	1	0	0	3	1564
18	Micrasema (gelidum) gentile McLachlan, 1880	EP+NA	1	0	0	0	0	0	0	0	0	0	1	4
Glossosomatidae														
19	*Agapetusbidens* McLachlan, 1875	EP+WP	0	0	1	0	0	0	0	1	0	0	2	165
20	*Agapetusjakutorum* Martynov, 1934	EP	1	0	0	0	1	0	0	0	0	0	2	151
21	*Agapetussibiricus* Martynov, 1918	EP	1	0	0	0	0	0	0	1	0	0	2	54
22	*Glossosomaaltaicum* (Martynov, 1914)	EP+WP	1	0	0	0	0	0	0	1	0	0	2	104
23	*Glossosomaangaricum* (Levanidova, 1967)	EP	1	0	0	0	0	0	1	0	0	0	2	2
24	*Glossosomaintermedium* (Klapálek, 1892)	HL	1	1	1	1	0	0	1	1	1	0	7	1375
25	*Glossosomanylanderi* McLachlan, 1879	EP+WP	1	0	1	1	1	0	0	1	1	0	6	176
26	*Glossosomaussuricum* (Martynov, 1934)	EP	1	0	0	0	0	0	0	0	0	0	1	1
27	*Glossosomaschmidi* Levanidova, 1979	EP	1	0	0	0	0	0	0	0	0	0	1	1
28	*Paduniaadelungi* Martynov, 1910	EP	1	0	0	0	1	0	0	1	0	0	3	817
29	*Paduniabikinensis* Martynov, 1934	EP	1	0	0	1	1	0	0	0	0	0	3	988
30	*Paduniaforcipata* Martynov, 1934	EP	0	0	0	0	0	1	0	0	0	0	1	1
31	*Synagapetusinaequispinosus* (Schmid, 1970)	EP	1	0	1	0	0	0	0	1	0	0	3	558
Goeridae														
32	*Archithremmaulachensis* Martynov, 1935	EP	1	0	0	0	0	0	0	0	0	0	1	3
33	*Goerajaponica* Banks, 1906	EP	1	0	1	0	1	1	0	1	0	0	5	108
34	*Goeratungusensis* Martynov, 1909	EP+NA	1	1	0	1	1	0	1	1	1	0	7	2968
Hydropsychidae														
35	*Aethalopteraevanescens* (McLachlan, 1880)	EP	0	1	0	0	0	0	0	0	0	0	1	1
36	*Arctopsycheamurensis* Martynov, 1934	EP	1	1	0	0	0	0	0	0	0	0	2	90
37	*Arctopsycheladogensis* (Kolenati, 1859)	HL	1	1	0	0	1	0	0	1	0	0	4	56
38	*Arctopsychepalpata* Martynov, 1934	EP	0	0	0	0	0	0	1	0	0	0	1	1
39	*Cheumatopsychecapitella* (Martynov, 1927)	EP+WP	1	0	0	0	0	1	0	0	0	0	2	3
40	**Cheumatopsycheinfascia* Martynov, 1934	EP+OL	0	0	0	0	0	1	0	0	0	0	1	13
41	*Hydropsycheangustipennis* (Curtis, 1834)	EP+WP	0	0	0	0	0	0	1	0	0	0	1	1
42	*Hydropsychebulgaromanorum* Malicky, 1977	EP+WP	1	0	0	0	0	0	0	0	0	0	1	110
43	*Hydropsychecontubernalis* McLachlan, 1865	EP+WP	1	0	0	0	0	0	0	0	0	0	1	227
44	*Hydropsychekozhantschikovi* Martynov, 1924	EP	1	1	1	0	0	0	1	1	1	0	6	2295
45	*Hydropsychelianchiensis* (Li & Tian, 1990)	EP	0	0	1	1	0	0	0	1	0	0	3	3
46	*Hydropsychenewae* Kolenati, 1858	EP+WP	1	1	1	1	1	1	1	1	1	0	9	1394
47	*Hydropsycheorientalis* Martynov, 1934	EP+OL	0	0	0	0	0	1	0	0	0	0	1	1
48	*Hydropsychepellucidula* (Curtis, 1834)	HL	0	0	1	0	0	0	0	0	0	0	1	103
49	*Hydropsychevalvata* Martynov, 1927	EP+OL	1	1	1	0	0	1	0	1	0	0	5	1489
50	*Macrostemumradiatum* (McLachlan, 1872)	EP+OL	1	0	0	0	0	0	0	0	0	0	1	2
51	*Potamyiaczekanovskii* (Martynov, 1910)	EP	1	0	1	1	1	0	0	1	0	0	5	926
Hydroptilidae														
52	*Agrayleamultipunctata* Curtis, 1834	HL	1	0	0	0	0	0	1	1	0	0	3	41
53	*Hydroptilaangulata* Mosely, 1922	EP+WP	1	0	1	0	1	0	1	1	0	0	5	206
54	*Hydroptilatineoides* Dalman, 1819	EP+WP	1	0	0	0	0	0	0	0	0	0	1	1
55	***Hydroptilapectinifera* Schmid, 1970	EP	1	0	0	0	0	0	0	0	0	0	1	1
56	*Oxyethiraecornuta* Morton, 1893	HL	1	0	0	0	0	0	0	0	0	0	1	2
57	*Oxyethiraflavicornis* (Pictet, 1834)	EP+WP	1	0	0	0	0	0	0	1	0	0	2	253
58	*Stactobiamakartschenkoi*Botosaneanu & Levanidova, 1988	EP	1	0	0	0	0	0	0	0	0	0	1	2
59	*Stactobiellaalasignata* Botosaneanu, 1993	EP	1	0	0	0	0	0	0	0	0	0	1	2
60	*Stactobiellabiramosa* Martynov, 1929	EP	1	0	0	0	0	0	0	0	0	0	1	1
Lepidostomatidae														
61	*Lepidostomaalbardanum* (Ulmer, 1906)	EP	1	0	0	0	1	0	0	0	0	0	2	74
62	*Lepidostomahirtum* (Fabricius, 1775)	EP+WP	1	0	0	0	1	0	0	1	0	0	3	91
63	*Lepidostomapenicillatum* (McLachlan, 1875)	EP+WP	0	0	1	0	0	0	0	1	0	0	2	50
64	*Lepidostomachaldyrense* (Martynov, 1909)	EP+WP	1	0	0	0	0	0	0	0	0	0	1	1
65	*Lepidostomastubbei* (Mey, 1980)	EP	0	0	0	0	0	0	0	1	0	0	1	1
Leptoceridae														
66	*Ceracleaalbimacula* (Rambur, 1842)	EP+WP	1	0	0	0	0	0	0	1	0	0	2	29
67	*Ceracleaannulicornis* (Stephens, 1836)	HL	1	0	1	1	0	1	0	1	0	0	5	217
68	*Ceracleaexcisa* (Morton, 1904)	HL	1	1	0	0	1	0	1	1	0	0	5	292
69	*Ceracleafulva* (Rambur, 1842)	EP+WP	1	0	0	0	0	0	1	0	0	0	2	98
70	*Ceracleaglobosa* Yang & Morse, 1988	EP	1	0	0	0	0	0	0	0	0	0	1	3
71	*Ceracleahastata* (Botosaneanu, 1970)	EP	1	0	0	0	0	0	0	0	0	0	1	1
72	*Ceraclealobulata* (Martynov, 1935)	EP	1	0	0	1	0	0	0	1	0	0	3	94
73	*Ceracleanigronervosa* (Retzius, 1783)	HL	1	1	1	0	0	0	0	1	0	0	4	140
74	*Ceracleashuotsuensis* (Tsuda, 1942)	EP	1	0	0	0	0	0	0	1	0	0	2	115
75	*Ceracleasibirica* (Ulmer, 1906)	EP	1	0	1	1	1	0	0	0	0	0	4	139
76	*Ceracleatrilobulata* Morse, Yang, & Levanidova, 1997	EP	1	0	0	0	0	0	0	0	0	0	1	1
77	*Erotesisbaltica* McLachlan, 1877	EP	0	0	0	0	0	0	1	0	0	0	1	1
78	*Mystacidesbifidus* Martynov, 1924	EP	1	0	0	0	0	0	0	0	0	0	1	1
79	*Mystacidesinterjectus* (Banks, 1914)	EP+NA	1	0	0	0	0	0	0	0	0	0	1	1
80	*Mystacideslongicornis* (Linnaeus, 1758)	EP+WP	1	0	0	1	0	0	0	1	0	0	3	518
81	*Mystacidessepulchralis* (Walker, 1852)	EP+NA	1	1	0	0	0	0	0	0	0	0	2	70
82	*Mystacidessibiricus* Martynov, 1935	EP+OL	0	0	0	0	0	0	1	0	0	0	1	1
83	*Oecetisfurva* (Rambur, 1842)	EP+WP	0	0	0	0	1	0	1	1	0	0	3	76
84	*Oecetisintima* McLachlan, 1877	EP+WP	1	0	0	1	0	0	0	1	0	0	3	43
85	*Oecetislacustris* (Pictet, 1834)	HL	1	1	0	0	0	0	1	1	0	0	4	38
86	**Oecetisnigropunctata* Ulmer, 1908	EP+OL	0	0	0	1	0	0	0	0	0	0	1	5
87	*Oecetisochracea* (Curtis, 1825)	HL	1	1	0	1	0	1	1	1	1	1	8	258
88	*Parasetodesaquilonius* Yang & Morse, 1997	EP	1	0	0	1	0	0	0	0	0	0	2	4
89	*Parasetodesrespersellus* (Rambur, 1842)	HL	0	0	0	0	0	1	0	0	0	0	1	2
90	*Setodesfurcatulus* Martynov, 1935	EP	0	0	0	0	1	0	0	0	0	0	1	1
91	*Setodespunctatus* (Fabricius, 1793)	EP+WP	1	0	0	1	0	0	0	1	0	0	3	345
92	*Triaenodesfulvus* Navas, 1931	EP+OL	1	0	0	0	0	0	0	0	0	0	1	1
93	*Triaenodesinternus* McLachlan, 1875	EP+WP	1	0	1	1	0	0	1	1	0	0	5	100
94	*Triaenodesjakutanus* Martynov, 1910	EP+NA	0	0	0	1	0	0	0	1	0	0	2	3
95	*Triaenodeslevanidovae* (Morse & Vshivkova, 1997)	EP	1	0	0	1	1	0	1	0	0	0	4	187
96	*Triaenodesreuteri* McLachlan, 1880	EP+WP	1	1	1	1	0	1	1	1	0	0	7	138
97	*Triaenodessimulans* Tjeder, 1929	EP+WP	1	0	0	1	1	0	0	0	0	0	3	123
Limnephilidae														
98	*Anaboliaappendix* (Ulmer, 1905)	EP	1	0	1	0	1	0	0	1	1	0	5	119
99	*Anaboliaservata* (McLachlan, 1880)	EP	1	0	0	1	0	0	0	0	0	0	2	2
100	*Anisogamodesflavipunctatus* (Martynov, 1914)	EP	1	0	0	0	1	0	1	1	0	0	4	93
101	*Annitellaobscurata* (McLachlan, 1876)	EP+WP	1	0	0	0	0	0	0	0	0	0	1	3
102	*Arctoporatrimaculata* (Zetterstedt, 1840)	HL	1	0	0	0	0	0	0	1	0	0	2	9
103	*Asynarchusamurensis* (Ulmer, 1905)	EP+WP	1	0	0	1	1	0	0	1	0	0	4	7
104	*Asynarchusiteratus* McLachlan, 1880	EP+NA	1	1	0	0	0	0	0	1	0	0	3	658
105	*Asynarchuslapponicus* (Zetterstedt, 1840)	HL	1	1	0	0	0	0	0	1	0	0	3	19
106	**Asynarchussachalinensis* Martynov, 1914	EP	1	0	0	0	0	0	0	0	0	0	1	1
107	*Asynarchusthedenii* (Wallengren, 1879)	EP+WP	1	0	0	0	0	0	0	0	0	0	1	2
108	*Brachypsycherara* (Martynov, 1914)	EP	1	0	0	0	0	0	0	0	0	0	1	2
109	*Chaetopteryxsahlbergi* McLachlan, 1876	EP+WP	1	0	0	0	0	0	0	0	0	0	1	1
110	*Clostoeca* sp	EP+NA	1	0	0	0	0	0	0	0	0	0	1	1
111	*Colpotauliusincisus* (Curtis, 1834)	EP+WP	1	1	0	1	0	1	1	1	0	1	7	86
112	*Dicosmoecuspalatus* (McLachlan, 1872)	EP+WP	1	1	1	1	0	0	1	1	0	0	6	298
113	**Drusus* sp	EP+WP	0	0	1	0	0	0	0	1	0	0	2	98
114	*Ecclisomyiadigitata* (Martynov, 1929)	EP	1	1	0	0	0	0	1	1	0	0	4	69
115	*Ecclisomyiakamtshatica* (Martynov, 1914)	EP	1	0	0	0	0	0	0	0	0	0	1	16
116	*Grammotauliussibiricus* McLachlan, 1874	EP+WP	1	0	0	0	0	0	0	0	0	0	1	1
117	*Grammotauliussignatipennis* McLachlan, 1876	HL	0	0	0	0	0	0	0	1	0	0	1	11
118	*Halesussachalinensis* Martynov, 1914	EP	1	0	0	0	0	0	0	1	0	0	2	6
119	*Halesustessellatus* (Rambur, 1842)	EP+WP	0	0	0	0	0	0	1	0	0	0	1	1
120	*Hydatophylaxgrammicus* (McLachlan, 1880)	EP+WP	1	1	1	0	0	0	0	0	0	0	3	25
121	*Hydatophylaxfestivus* (Navas, 1920)	EP	1	0	0	0	0	0	0	0	0	0	1	1
122	*Hydatophylaxnigrovittatus* (McLachlan, 1872)	EP	1	1	0	0	0	0	0	0	0	0	2	57
123	*Hydatophylaxsoldatovi* (Martynov, 1914)	EP	1	0	0	0	0	0	0	0	0	0	1	10
124	*Hydatophylaxvariabilis* (Martynov, 1910)	HL	1	0	0	0	0	0	0	0	0	0	1	1
125	*Lenarchusproductus* (Morton, 1896)	EP+WP	0	1	0	0	0	0	0	0	0	0	1	2
126	*Lepnevainasignata* Wiggins, 1987	EP	0	0	0	0	0	0	0	1	0	0	1	1
127	*Limnephilusabstrusus* McLachlan, 1872	EP	1	0	0	1	0	0	1	0	0	0	3	7
128	*Limnephilusalaicus* (Martynov, 1915)	EP+WP	0	0	0	0	0	0	1	1	1	0	3	12
129	*Limnephilusalgosus* (McLachlan, 1868)	HL	1	0	0	0	0	0	1	1	0	1	4	122
130	*Limnephilusasiaticus* (McLachlan, 1874)	HL	1	0	0	0	0	0	1	1	0	0	3	8
131	*Limnephilusbulgani* Mey, 1991	EP	1	0	0	0	0	0	0	0	0	0	1	1
132	*Limnephiluscorreptus* McLachlan, 1880	EP	1	0	0	0	0	0	0	0	0	0	1	1
133	*Limnephilusdiphyes* McLachlan, 1880	HL	1	0	0	0	0	0	0	0	0	0	1	1
134	*Limnephilusdispar* McLachlan, 1875	HL	1	0	0	0	0	0	0	0	0	0	1	7
135	*Limnephilusextricatus* McLachlan, 1865	EP+WP	1	1	0	0	0	0	1	0	0	0	3	40
136	*Limnephilusflavicornis* (Fabricius, 1787)	EP+WP	1	0	0	0	0	0	0	0	0	0	1	1
137	*Limnephilusfenestratus* (Zetterstedt, 1840)	HL	1	0	0	0	0	0	0	1	0	0	2	7
138	*Limnephilusfuscinervis* (Zetterstedt, 1840)	EP+WP	0	0	1	0	0	0	0	0	0	0	1	1
139	*Limnephilusfuscovittatus* Matsumura, 1904	EP+OL	1	1	0	0	0	0	1	1	0	0	4	55
140	***Limnephilushovsgolicus* Morse, 1999	EP	1	0	0	0	0	0	0	0	0	0	1	42
141	*Limnephilusmajor* (Martynov, 1909)	HL	1	0	0	0	0	0	1	1	0	0	3	11
142	*Limnephiluspicturatus* McLachlan, 1875	HL	1	1	0	0	0	0	0	1	0	0	3	8
143	*Limnephiluspolitus* McLachlan, 1865	EP+WP	1	1	0	0	0	0	0	0	0	0	2	2
144	*Limnephilusprimoryensis* Nimmo, 1995	EP	0	0	0	0	0	0	1	0	0	1	2	35
145	*Limnephilusquadratus* Martynov, 1914	EP+WP	1	0	0	0	0	0	0	0	0	0	1	1
146	*Limnephilusrhombicus* (Linnaeus, 1758)	HL	1	1	0	1	1	0	1	0	0	0	5	98
147	*Limnephilussamoedus* (McLachlan, 1880)	HL	0	0	1	0	0	0	0	1	0	0	2	8
148	*Limnephilussparsus* Curtis, 1834	EP+WP	0	1	0	0	0	0	1	0	0	1	3	113
149	*Limnephilusstigma* Curtis, 1834	HL	1	1	0	0	0	0	0	1	0	0	3	44
150	*Limnephilussubnitidus* McLachlan, 1875	EP+WP	1	0	0	0	0	0	0	1	0	0	2	98
151	*Limnephilusvittatus* (Fabricius, 1798)	EP+WP	0	1	0	0	0	0	0	0	0	0	1	1
152	*Micropternasequax* McLachlan, 1875	EP+WP	1	0	0	0	0	0	0	0	0	0	1	2
153	*Nemotauliusadmorsus* (McLachlan, 1866)	EP	1	0	0	0	0	0	0	0	0	0	1	4
154	*Nemotauliusamurensis* Nimmo, 1995	EP	1	1	0	0	0	0	0	0	0	0	2	19
155	*Nemotauliusmutatus* (McLachlan, 1872)	HL	1	0	0	0	0	0	0	0	0	0	1	1
156	*Philarctusbergrothi* McLachlan, 1880	HL	1	1	0	0	0	1	0	1	0	0	4	109
157	*Philarctusrhomboidalis* Martynov, 1924	EP+WP	1	0	0	0	1	0	0	1	0	0	3	3
158	*Potamophylax* sp	EP+WP	1	0	0	0	0	0	0	0	0	0	1	2
159	*Pseudostenophylax* sp	HL	1	0	0	0	0	0	0	0	0	0	1	3
Molannidae														
160	*Molannaalbicans* (Zetterstedt, 1840)	EP+WP	0	0	0	0	0	0	0	1	0	0	1	200
161	*Molannamoesta* Banks, 1906	EP+OL	0	0	0	0	1	0	0	0	0	0	1	1
162	*Molannasubmarginalis* McLachlan, 1872	EP+WP	1	1	0	0	0	0	0	0	0	0	2	15
163	*Molannodestinctus* (Zetterstedt, 1840)	HL	1	0	0	0	1	0	1	1	0	0	4	79
Phryganeidae														
164	*Agrypniacolorata* Hagen, 1873	HL	1	0	0	1	0	0	0	1	0	0	3	19
165	*Agrypniacrassicornis* (McLachlan, 1876)	EP+WP	1	0	0	0	0	0	1	1	0	0	3	19
166	*Agrypniaczerskyi* (Martynov, 1924)	EP+WP	1	0	0	1	1	0	1	0	0	0	4	32
167	***Agrypniahayfordae* Morse & Chuluunbat, 2007	EP	1	0	0	0	0	0	0	0	0	0	1	339
168	*Agrypniaobsoleta* (Hagen, 1864)	HL	1	1	1	1	0	0	0	1	0	0	5	345
169	*Agrypniapagetana* Curtis, 1835	HL	1	1	1	0	0	0	1	1	0	0	5	12
170	*Agrypniapicta* Kolenati, 1848	EP+WP	1	1	0	1	0	1	1	0	0	0	5	40
171	*Hagenella* sp	HL	1	0	0	0	0	0	0	0	0	0	1	1
172	*Oligotrichahybridoides* Wiggins & Kuwayama, 1971	EP	1	0	0	0	0	0	0	0	0	0	1	1
173	*Oligotrichalapponica* (Hagen, 1864)	HL	1	1	0	0	0	0	0	0	0	0	2	7
174	*Oligotrichastriata* (Linnaeus, 1758)	EP+WP	1	0	0	0	0	0	0	0	0	0	1	1
175	*Phryganeabipunctata* Retzius, 1783	EP+WP	1	1	0	0	1	0	0	1	0	0	4	5
176	*Phryganeagrandis* Linnaeus, 1758	EP+WP	1	1	0	0	0	0	1	1	0	0	4	25
177	*Semblisatrata* (Gmelin, 1789)	EP+WP	1	0	0	0	0	0	0	1	0	0	2	192
178	*Semblisphalaenoides* (Linnaeus, 1758)	EP+WP	1	0	0	0	0	0	0	0	0	0	1	1
Polycentropodidae														
179	*Cyrnus* sp	EP+WP	0	0	0	0	0	0	1	0	0	0	1	1
180	*Holocentropuspicicornis* (Stephens, 1836)	HL	1	0	0	0	0	0	0	0	0	0	1	2
181	*Neucentropusmandjuricus* (Martynov, 1907)	EP+OL	0	0	0	0	0	1	0	0	0	0	1	1
182	*Neureclipsisbimaculata* (Linnaeus, 1758)	HL	0	1	0	0	0	0	0	1	0	0	2	31
183	**Nyctiophylax* sp	HL	0	0	0	0	0	0	1	0	0	0	1	1
184	*Plectrocnemiakusnezovi* Martynov, 1934	EP	1	0	0	0	0	0	0	0	0	0	1	1
185	*Polycentropusflavomaculatus* (Pictet, 1834)	EP+WP	0	0	0	0	0	0	0	1	0	0	1	2
Psychomyiidae														
186	*Psychomyiaflavida* Hagen, 1861	EP+NA	1	0	1	1	1	1	1	1	1	0	8	7739
187	*Psychomyiaminima* (Martynov, 1910)	EP	1	0	1	0	1	0	0	1	1	0	5	1565
188	*Psychomyiapusilla* (Fabricius, 1781)	EP+WP	1	0	0	0	0	0	0	0	0	0	1	1
Rhyacophilidae														
189	*Himalopsyche* sp	EP+NA+OL	1	0	0	0	0	0	0	0	0	0	1	1
190	*Rhyacophilaangulata* Martynov, 1910	EP+OL	1	0	1	0	1	0	0	1	0	0	4	329
191	*Rhyacophiladepressa* Martynov, 1910	EP	1	0	0	0	1	0	0	0	0	0	2	23
192	*Rhyacophilaegijnica* Schmid, 1968	EP	1	1	1	1	1	0	1	1	1	0	8	3349
193	*Rhyacophilaimpar* Martynov, 1914	EP	1	0	0	0	1	0	0	1	0	0	3	7
194	*Rhyacophilalata* Martynov, 1918	EP	1	0	0	0	0	0	0	0	0	0	1	76
195	*Rhyacophilamongolica* Levanidova, 1993	EP	1	1	0	0	0	0	0	0	0	0	2	77
196	*Rhyacophilanana* Levanidova, 1993	EP	1	0	0	0	0	0	0	0	0	0	1	8
197	*Rhyacophilanipponica* Navas, 1933	EP	1	0	0	0	0	0	0	0	0	0	1	1
198	*Rhyacophilaretracta* Martynov, 1914	EP	1	0	0	0	0	0	0	0	0	0	1	11
199	*Rhyacophilasibirica* McLachlan, 1879	EP	1	1	1	1	0	0	1	1	0	0	6	411
Stenopsychidae														
200	*Stenopsychegriseipennis* McLachlan, 1866	EP+OL	0	0	1	0	0	1	0	0	0	0	2	14
Thremmatidae														
201	*Neophylax* sp	EP+NA+OL	1	0	0	0	0	0	0	0	0	0	1	1
TOTAL			159	51	39	38	34	17	52	87	15	7	499	47939
